# Bird Communities and Environmental Correlates in Southern Oregon and Northern California, USA

**DOI:** 10.1371/journal.pone.0163906

**Published:** 2016-10-12

**Authors:** Jaime L. Stephens, Eric C. Dinger, John D. Alexander, Sean R. Mohren, C. John Ralph, Daniel A. Sarr

**Affiliations:** 1 Klamath Bird Observatory, Ashland, Oregon, United States of America; 2 National Park Service, Klamath Network, Ashland, Oregon, United States of America; 3 Crater Lake National Park, Oregon, United States of America; 4 USDA Forest Service, Pacific Southwest Research Station, Redwood Sciences Laboratory, Arcata, California, United States of America; Chinese Academy of Forestry, CHINA

## Abstract

We examined avian community ecology in the Klamath Ecoregion and determined that individual bird species co-exist spatially to form 29 statistically distinguishable bird groups. We identified climate, geography, and vegetation metrics that are correlated with these 29 bird groups at three scales: Klamath Ecoregion, vegetation formation (agriculture, conifer, mixed conifer/hardwood, shrubland), and National Park Service unit. Two climate variables (breeding season mean temperature and temperature range) and one geography variable (elevation) were correlated at all scales, suggesting that for some vegetation formations and park units there is sufficient variation in climate and geography to be an important driver of bird communities, a level of variation we expected only at the broader scale. We found vegetation to be important at all scales, with coarse metrics (environmental site potential and existing vegetation formation) meaningful across all scales and structural vegetation patterns (e.g. succession, disturbance) important only at the scale of vegetation formation or park unit. Additionally, we examined how well six National Park Service units represent bird communities in the broader Klamath Ecoregion. Park units are inclusive of most bird communities with the exception of the oak woodland community; mature conifer forests are well represented, primarily associated with conifer canopy and lacking multi-layered structure. Identifying environmental factors that shape bird communities at three scales within this region is important; such insights can inform local and regional land management decisions necessary to ensure bird conservation in this globally significant region.

## Introduction

In any given landscape, a hierarchy of factors influence individuals, populations, and communities [1]. The study of community ecology integrates the occurrence, co-occurrence, and interactions of species with their measurable environment [2–4]. Bird distribution studies have emphasized identification of functional [5,6], taxonomic [7], or habitat specific [8] groups. Community delineation is problematic because the full extent of components and interactions can never be fully known, hence classifications are arbitrary [9], and even where clear units are recognizable at a point in time, they may not coexist and thus interact over time [10]. Despite this, improved understanding of distributional dynamics and co-occurrence of taxa have strong implications for biodiversity conservation [11].

We define bird communities, as others have, as individual suites of species co-occurring on the landscape at a given time [2,3]. Bird community patterns are the result of species composition and distribution across spatial and temporal gradients [12]. These ecological patterns are scale dependent, with influences ranging from broad to local scales. An individual species has ecological tolerances defining what environment it can reside in, and the spatial existence of those conditions determines where a species can exist, defining the potential species range and limiting potential community members at a locality [3]. Range boundaries can be further influenced by habitat suitability, resource availability, dispersal, and competition [3] some of the same factors that influence species occurrence at the local scale. Within their geographic range, species distributions are often patchy due to the influence of local habitat conditions in determining where individual birds establish breeding territories and nest sites [3]. These decisions are based largely on nest site availability and predator avoidance, both influenced by vegetation structure and floristics and related to topography, physiognomy, and climate [2,13]. Thus, factors influencing bird communities at a given location result from gradients of spatial scales interacting with a hierarchical organization of environmental tolerances and choices [14].

The Klamath Ecoregion extends across nearly 17.5 million hectares from the central Pacific coast of North America, over several mountain ranges, east to the Great Basin. It is an area with complex geology and climate, and correspondingly high plant diversity and biogeographic centrality [15–18]. These same factors make this region significant for avian biodiversity and biogeography [19,20]. Early studies of Klamath bird distributions suggest that it is a crossroads in avian biogeography and rich in species diversity [19,21]. In the early 1900s Anderson and Grinnell [21] noted how the western Siskiyou Mountains formed a narrow line of tension between coastal and Sierran species. A recent study documented distribution limits for 37 species within this region; 18 reaching a western limit, 8 a northern limit, 3 northern and eastern limits, and 7 a southern limit [19]. Udvardy [22] describes a high number of biogeographic units in the relative small area of the Klamath Ecoregion, including Oregonian, Californian, Great Basin, and Cascade-Sierran provinces within their Nearctic Realm, consistent with the transitional nature of the avifauna.

A diversity of land owners and associated land use and management practices are prevalent in the Klamath Ecoregion [23], including 57% federally owned, with 13% of that located in six National Park Service units (parks, monuments and recreation areas). National Park Service lands are dedicated to “conserve the scenery and the natural and historic objects and the wildlife therein and to provide for the enjoyment of the same in such manner and by such means as will leave them unimpaired for the enjoyment of future generations” [24]. Although early management focused on excluding logging, grazing, and mining to maintain “everlasting wildness” [25], scientific studies of California national parks in the 1930s showed declines in native species (especially predators), exotic plant and animal introductions, and road impacts [25]. Despite challenges of balancing recreation and preservation mandates [25], the National Park Service is considered an agency that manages a major part of the protected land area of the United States [26], although such protected areas typically fall short of capturing the full array of biodiversity in any region [27].

We examine community ecology in the Klamath Ecoregion to determine whether individual bird species co-exist spatially to form bird communities and if so, to identify gradients of climate, geography, and vegetation associated with those communities. Because patterns and processes shaping bird communities are scale dependent, we consider three spatial scales: Klamath Ecoregion, vegetation formation, and National Park Service unit. We test the hypotheses that 1) compositionally distinct bird groups can be identified in the Klamath Ecoregion and if those groups exist, 2) environmental factors including a number of climate, geography, and vegetation variables will relate differently at the three spatial scales (Klamath Ecoregion, vegetation formation, National Park Service unit). We suggest that correlations with climate variables will be most important at the ecoregion scale, geography variables most important at the vegetation formation scale, and vegetation variables most important at the park unit scale.

Previous work has examined bird communities in adjacent regions, e.g. Oregon Coast Range [14] and Great Basin shrub-steppe [28], but to our knowledge no such multi-scale study has occurred in the Klamath Ecoregion. Identifying environmental factors that shape bird communities at three scales within this region is important because local and regional land management decisions, including management of altered disturbance regimes, are applied at those scales. Additionally, through this scaled approach, we aim to identify how well the National Park Service units represent the composition and diversity of bird communities in this region. In combination, we aspire to inform conservation efforts for both birds and the habitats that they represent.

## Methods

### Study Area

**Fig 1:**
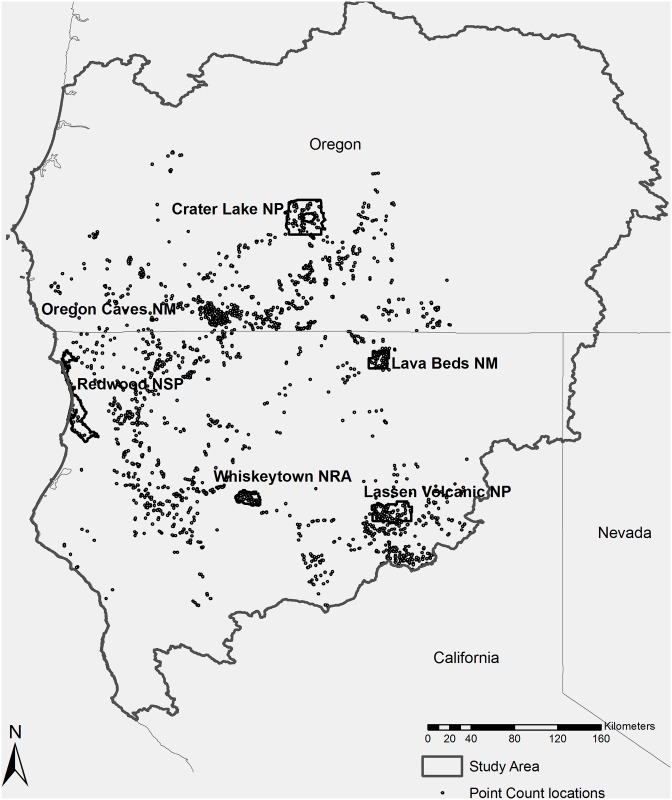
Study Area. The study area is defined by the Klamath Ecoregion located in northern California and southern Oregon. Final analyses included 1990 bird survey points (see methods); 259 were located within the six National Park Service park units.

The Klamath Ecoregion is located in northern California and southern Oregon. This region has been described slightly differently by various authors [17,29,30]; here, we define it as the Klamath, Rogue, Umpqua, and Lassen HUC-8 watersheds (Fig 1). It extends from the Pacific Ocean to the east slope of the Cascade Mountains and adjacent volcanic plateaus, with average elevation in the mountains ranging from 1500–2000 meters. The biogeography of this region is largely influenced by proximity to the ocean and the convergence of the Cascade, Siskiyou, and Klamath Mountains. The Klamath Ecoregion is the meeting point for several biogeographic provinces and associated ecoregions: California coast range, Oregon coast range, Cascade range, California central valley, Sierra Nevada range, and Great Basin [15,30]. The region has a varied climate, primarily influenced by distance from ocean, elevation, and latitude [19].

The varied geology, soil, and climate, combined with correspondingly heterogeneous disturbance histories, contribute to diverse vegetation communities [31]. The region is dominated by coniferous and mixed conifer hardwood forests, with important components of grassland, meadow, chaparral, and shrub-steppe. The western edge of the region is dominated by redwood forests (*Sequoia sempervirens)* with spruce *(Picea sutchensis)* and western hemlock *(Tsuga heterophylla)* in the northern parts. Inland, the western part of the region is dominated by mixed conifer forests largely composed of Douglas fir (*Pseudotsuga menziesii*) with hardwood components; high elevation sites, with significant winter snowpack, are dominated by mixed conifer forests with diverse assemblages of conifer species, and at the highest elevations by true fir (*Abies* spp.), mountain hemlock (*Tsuga mertensiana*), and whitebark pine (*Pinus albicaulis*). Oak (*Quercus* spp.) woodlands, grasslands and chaparral are intermixed throughout the forest stands, increasing in importance from west to east, with the easternmost edge predominately shrub-steppe. Riparian vegetation is also found throughout the region including willows (*Salix* spp.), alders (*Alnus* spp.), and at higher elevations wet alpine meadows and mesic aspen (*Populus tremuloides*) stands [31,32].

The study area includes six National Park Service units: Crater Lake National Park (Crater Lake NP), Lassen Volcanic National Park (Lassen Volcanic NP), Lava Beds National Monument (Lava Beds NM), Oregon Caves National Monument (Oregon Caves NM), Redwood National and State Parks (Redwood NSP), and Whiskeytown National Recreation Area (Whiskeytown NRA). The park units are broadly distributed across the Klamath Ecoregion and generally include the habitat types described above (Fig 1). Research permits were obtained from all National Park Service units.

### Sampling Design

#### Avian data

We used existing avian data collected within the Klamath Ecoregion from 1992 to 2013. Data were collected following point count methodology, which is the standard for counting birds on their breeding grounds where they have established territories and sing regularly throughout the nesting period [33]. Surveys were conducted by Klamath Bird Observatory, Point Blue Conservation Science, and U.S. Forest Service Redwood Sciences Laboratory in partnership with other federal agencies and nongovernmental organizations. Point count surveys occurred during the bird breeding season, primarily between mid-May and late June, with surveys in early May at low elevation areas of the study area’s southern extent and later through mid-July at the highest elevation sites. All surveys began within 15 minutes of sunrise, were completed within four hours, and not conducted in inclement weather. All birds detected by sight or sound during a five minute period were identified to species. For analyses, we included only species well surveyed by this methodology, i.e., songbirds (passerines), woodpeckers, and hummingbirds (S1 Table). Individuals flying over but not using the area were excluded. Because at relatively large geographic scales distributional patterns are typically determined by presence/absence, and due to the nature of the survey data (heavily influenced by zeros with counts generally ranging from one to two individuals), we reduced our dataset to presence/absence.

The dataset included 19,395 sites where point count surveys were completed; 2528 sites were located in National Park Service units. For sites visited in multiple years, we used the most recent survey date. To generate a random sample from the available data we used a Generalized Random Tessellation Stratified (GRTS) [34] algorithm to generate 2000 spatially balanced subsamples. GRTS is a methodological way of selecting a subset of sites that maximizes spatial spread and minimizes spatial autocorrelation [35], by using reverse hierarchical ordering so that any resulting contiguous list of sites is spatially balanced.

#### Environmental variables

We used spatial datasets from five landscape scale sampling or modeling programs to develop 14 continuous and eight categorical environmental variables for our multi-scale statistical analyses [36–43]. We selected metrics that had the potential to correlate with bird communities, including climate, geography, and vegetation variables (S2 Table).

April through July climate data from the PRISM Climate Group were selected to determine 30-year breeding season average temperature and precipitation ‘normals’ [33,36,43,44]. Normals are baseline datasets at an 800m spatial resolution that describe the average monthly conditions over the timespan [43] (S2 Table).

Geographic data were derived from various datasets. We calculated distance to roads from the National Park Service NPScape landscape dynamics monitoring project [39,41]. We used NHDFlowline and NHDwaterbody hydrography data from National Hydrography Dataset (NHD) to determine nearest distance to perennial streams and all streams, distance to the coastline, and nearest distance to any waterbody (e.g., lakes, ponds, marsh) [45]. Topographical variables, including aspect, slope, and elevation, were generated from the Landscape Fire and Resource Management Planning Tools [42] (S2 Table).

We used LANDFIRE to generate datasets related to vegetation (existing vegetation height, type, and cover, biophysical settings, and environmental site potential parameters), fuels (forest canopy cover, forest canopy height, and forest canopy bulk density), and disturbance (succession classes) [42]. As measures of productivity, leaf area index (LAI) and Fraction of absorbed Photosynthetically Active Radiation (FPAR; hereafter vegetative productivity) were obtained at a 1000m spatial resolution from satellite imagery from the Land Processes Distributed Active Archive Center (LP DAAC) [37,38] (S2 Table).

### Analyses

#### SIMPROF

The number and composition of bird groups in the Klamath Ecoregion were determined using Similarity Profile Test (SIMPROF) in the multivariate analysis package Primer-E [46,47]. SIMPROF works as an iterative process, testing individual nodes of a cluster analysis (using Sørenson similarities and group average linkage method). At each node, SIMPROF tests for underlying structure. Samples below a node are ranked by similarity, forming a “similarity profile,” and a null model of no structure is derived by randomizing the allocation of species at a site, and repeating the rank profile (many times to develop a mean randomized profile). A departure statistic *π* is then calculated as the deviation of the observed profile against the mean randomized profile. A null distribution of π is then calculated by repeated permutation of the randomized site by species matrix. The observed departure statistic, π, can then be referred to the null distribution of π. The null hypothesis of no significant structure in that node can then be rejected using the standard permutation tests [48] at the level of P < (# observed greater or equal values of π + 1)/(# of randomizations + 1). All randomization procedures used in this manuscript used 999 randomizations.

SIMPROF’s advantage is that it is iterative, and provides a stopping rule for unwarranted analysis of the substructure of each node [47]. Additionally, each cluster is considered separately, so that an arbitrary amount of similarity is not used to determine ecological significance, allowing some clusters to have ecologically significant sub-groups while others have absence of structure. However, due to a large sample size, significance can occur with subtle biological similarities. To account for statistical significance interfering with ecological significance we selected a lower than typical (P < 5%); α level of 0.3% for rejecting the null hypothesis of no ecological structure. The end result of SIMPROF is an *a posteriori* bird grouping scheme derived from the species presence/absence data.

We associated each bird group with a single vegetation formation using two LANDFIRE variables. First, we used LANDFIRE EVT_PHYS to assign each group to a vegetation formation (agriculture, conifer forest, or shrubland) based on the formation most common among the sites that contributed to the SIMPROF group. This worked for all but one group, which had sites split evenly in conifer and shrubland. In that instance we looked at the finer resolution class LANDFIRE EVT_GP_N (Existing Vegetation Type Group Name), and because conifer included juniper, which is closely related to shrubland in this region, we classed this group as shrubland. To further parse out the conifer vegetation formation into pure conifer versus mixed conifer/hardwood we again used the finer resolution LANDFIRE class. Previous work has suggested 20–60% total cover comprised of broadleaf species to be indicative of mixed conifer/hardwood in our study area [13]. Following that, we assigned a species group as mixed conifer/hardwood if >20% of the sites had classifications of chaparral, conifer-oak forest and woodland, juniper woodland and savanna, aspen forest, woodland and parkland, or western oak woodland and savanna.

#### SIMPER

Species important in defining the bird groupings across the Klamath Ecoregion were determined using Similarity Percentage (SIMPER) routine in Primer-E. SIMPER works from groups (in this case determined using SIMPROF), and calculates the average similarity across all the samples within the group. In turn, it takes each bird species and leaves it out, and then recalculates the average similarity. The change in similarity is the contributing amount of the bird species removed. By repeating this procedure, the contribution of each bird species to group similarity is derived. A bird species was considered a “defining” species if it contributed to the top 90% of total group similarity.

#### Visualization of patterns

The graphic representation of how sites relate to one another in ecological space was viewed to discern finer scale relationships (i.e., plots of a single vegetation formation) using Non-metric Multidimensional Scaling (NMS) [49]. Once configured, sites can be identified based on park unit to gain insight into their relationship among the wider configuration of sites. Species with a correlation coefficient ≥ 0.5 are displayed.

#### Relating environmental variables to bird community plots

We explored the relationship of environmental metrics to the observed sites using three mechanisms: (1) Analysis of Similarity tests on *a priori* categorical environmental classes, (2) Biota-Environmental matching, where a subset of best performing continuous environmental variables was determined; and (3) correlation of continuous environmental variables with the axes of the NMS ordinations. Each is briefly described below.

Analysis of Similarity (ANOSIM) was used to determine linkages between categorical environmental variables (e.g., succession class, vegetation class) and sites [50]. ANOSIM is a multivariate test for the null hypothesis of no differences in bird groups between the classes within the variable. A test statistic, *R*, which reflects the average rank similarities within the classes versus average rank similarities between different classes is examined. *R* is closer to 1 if plots within the environmental classes are more similar to each other than any sites from other classes (i.e., complete separation of classes), and is close to 0 if the similarity is the same between and within classes (i.e., little or no segregation of classes). Significance can be attributed by permuting the class membership, creating a distribution of *R* under the null model of no differences. Because the large number of sites can create statistical significance despite only subtle biological significance, we used the *R* statistic as a measure of effect size for comparing the relative importance of categorical environmental variables against each other and across the multiple scales (Klamath Ecoregion, vegetation formation, park unit).

The best performing subset of continuous environmental variables was examined using the Primer-E routine Biota-Environment (BIOENV), either by looking at every possible combination of environmental variables or by a step-wise inclusion/elimination process where too many combinations existed. For these possible combinations, a Spearman’s ρ was calculated. The best performing set of environmental variables were those that maximize ρ, while minimizing the number of environmental variables. Significance of the best performing subset can be determined via a permutation test as described previously.

How these best fit environmental variables correlate with the bird community plots was visualized using exploratory vector overlays on the NMS ordinations, where the continuous environmental variable is correlated to the axes, with the overall direction and correlation strength being a function of the two axes (utilizing the Pythagorean Theorem). Although purely correlative, these biplots offer insight into the relationships between sites and environmental variables.

## Results

There were 96 songbird (passerine), woodpecker, and hummingbird species detected in the Klamath Ecoregion (S1 Table). The cluster analysis revealed 34 statistically distinct bird groups (Table 1). Each group was comprised of one (0.1%) to 577 (28.9%) sites. Five groups that were made up of only a single site or a single species were dropped; 29 groups (retaining 1990 of 2000 sites and all species) were included in further analyses. Average similarity within groups ranged from 26% to 47% (Table 1). The number of species making up individual groups ranged from 14 to 77, with 90% of the similarity within each group explained by two to 16 “defining” species. The average number of species per site within a group ranged from 3.6 to 9.5 (Table 1).

**Table 1:** Statistically Defined Bird Groups.

	Sampling Plots			Avg. Similarity	Total # Species	Avg # Species per Plot
Group	#	%	Defining Bird Species			
a	1	0.1%	*	*	*	*
b	1	0.1%	*	*	*	*
c	3	0.2%	*	*	*	*
d	7	0.4%	YRWA, GCKI, TOSO	29%	14	3.6
e	41	2.1%	STJA, HETH, BHGR, BTYW, SPTO	29%	47	4.3
f	10	0.5%	RBNU, OSFL, WREN, DEJU, CAVI	32%	17	3.9
g	18	0.9%	WETA, WEFL	47%	20	2.9
h	18	0.9%	FOSP, DUFL, DEJU, OSFL, WETA	39%	27	5.0
i	41	2.1%	DEJU, AMRO, STJA, NOFL	33%	53	4.3
j	7	0.4%	DEJU, HAFL, RBNU, SOSP, MGWA	44%	25	7.6
k	16	0.8%	GCKI, HETH, BRCR, DEJU, BHGR, RBNU, MOCH, HEWA	40%	28	6.9
l	54	2.7%	MOCH, DEJU, WEWP, AMRO, MGWA, WAVI, STJA, GCKI, WETA, YRWA, WIWA	40%	55	9.4
m	577	28.9%	RBNU, YRWA, DEJU, WETA, MOCH, HEWA, HETH, STJA, DUFL, AMRO	34%	77	7.2
n	71	3.6%	MOCH, YRWA, AMRO, CHSP, NOFL, WETA, WEWP, GRFL, TOSO, DUFL, HAWO, BHCO, DEJU	31%	59	7.6
o	6	0.3%	HOWR, BHGR, YRWA, TOSO, WBNU, STJA, WEWP	41%	20	7.2
p	35	1.8%	CAVI, BHGR, BTYW, DEJU, OCWA, CORA, WAVI	36%	43	6.2
q	17	0.9%	OSFL, STJA, RBNU, DUFL, PIWO, SPTO, NAWA, PUFI, WEWP, WAVI, WETA	38%	37	8.1
r	372	18.6%	WETA, BHGR, STJA, NAWA, DEJU, CAVI, WEFL, SPTO, RBNU, AMRO, HEWA, MGWA, WAVI	33%	76	8.7
s	146	7.3%	WETA, WEWP, BHGR, LAZB, DEJU, NOFL, STJA, SPTO, AMRO, LEGO, CHSP	33%	74	8.0
t	7	0.4%	BHCO, AMRO, HOFI, HOWR	35%	21	5.6
u	9	0.5%	ATFL, BBMA, BHCO, BUSH, BHGR	30%	23	4.8
v	35	1.8%	ATFL, SPTO, BHGR, EUST, HOWR, BUOR AMGO, HOFI, WESJ, BHCO, OJTI, WEWP, WEKI, DOWO, YBCH, WBNU	34%	45	9.5
w	41	2.1%	ACWO, OJTI, ATFL, LEGO, BEWR, WEME, WBNU, WEWP	41%	45	7.0
x	39	2.0%	WEWP, LAZB, HOWR, SPTO, AMRO, OSFL, LEGO, NAWA, CHSP, NOFL, STJA, WETA, RBNU	28%	65	7.8
y	113	5.7%	SOSP, WEWP, BHGR, YBCH, WEWA, SPTO, AMRO, WETA, OCWA, CAVI, WESJ, BUOR, WAVI	32%	71	8.4
z	95	4.8%	SPTO, BHGR, WREN, BEWR, NOFL, LAZB, OCWA, CHSP, NAWA, WETA, WESJ, AMRO, STJA, WEWP, BTYW	26%	74	6.9
aa	4	0.2%	*	*	*	*
ab	11	0.6%	WIWA SOSP STJA SWTH GCKI, MGWA	38%	20	4.8
ac	61	3.1%	WEFL, PAWR, CBCH, HEWA, VATH, WIWA, STJA, SWTH, WREN, AMRO, HUVI	27%	46	5.7
ad	38	1.9%	RWBL, SOSP, MAWR, SAVS, YHBL	46%	34	4.8
ae	1	0.1%	*	*	*	*
af	5	0.3%	WEWP, WEME, BBMA	33%	15	4.8
ag	67	3.4%	WEME, ROWR, SPTO, WESJ, BHCO, BEWR	34%	44	4.8
ah	33	1.7%	WEME, BRBL, AMRO, HOLA, VESP, MOCH, BRSP, SAVS	31%	35	5.1

Summary of statistically defined bird groups as determined with Similarity Profile (SimProf). “Group” is the arbitrary SimProf identifier. Defining bird species (see S1 Table for common and scientific names) are species contributing to 90% or greater of the overall group similarity (SIMPER), an asterisk indicates too few plots or species (<2) for inclusion in further analyses. Average similarity is average within group pair-wise Sørensen similarity. Total number of species and average number of species for all sites contributing to each group are provided. See methods for additional explanation.

### Klamath Ecoregion

Across the region, of eight categorical variables included in analyses the three broadest vegetation variables were most influential: environmental site potential, existing vegetation formation groups, and broad classification (Table 2). Bird groups were strongly related to continuous environmental variables (Table 3); six of these were best correlated including two climate, two geography, and two vegetation variables (Table 4).

**Table 2:** Grouping Strength for Categorical Environmental Variables.

Categorical Environmental Variable	Klamath Ecoregion	Vegetation Type	Park Unit	Significant Vegetation Types	Significant National Park Service Park Units
Environmental Site Potential Category	0.314	0.29775	0.263	All	CRLA, LABE, LAVO, REDW, WHIS
Existing Vegetation Type Groups	0.308	0.2055	0.2222	All	CRLA, LABE, LAVO, REDW, WHIS
Existing Vegetation Type Broad Classification	0.229	0.0795	0.1404	Conifer, Shrubland	LABE
Existing Vegetation Cover Category	0.178	0.1565	0.0858	All	CRLA
Existing Vegetation Type Class	0.166	0.11	0.1178	All	LABE, REDW
Succession Class Category	0.078	0.04725	0.1298	Conifer, Mixed Conifer/Hardwood, Shrubland	REDW
Aspect Categories	0.037	0.07575	0.0528	Agriculture, Conifer, Shrubland	LAVO, REDW
Disturbance Category	-0.03	-0.01475	0.0464	NONE	LABE, WHIS

Raw and averaged ANOSIM global R values for categorical environmental variables for grouping strength in the Klamath Ecoregion (raw), vegetation formation (average), and park unit (average). Significant vegetation formations and park units are those individual groups for which ANOSIM showed significant differences for that variable. See S2 Table for more information on environmental variables.

**Table 3:** Correlation Coefficients for Continuous Environmental Variables.

Continuous Environmental Variable	Klamath Ecoregion	Vegetation Formation				National Park Service Park Units				
		Shrubland	Agriculture	Conifer	Mixed Conifer/ Hardwood	Whiskey-town	Crater Lake	Lava Beds	Redwoods	Lassen Volcanic
km to Any Stream	0.37	0.49	0.46	0.34	0.25	0.41	0.15	0.56	0.07	0.37
km to Coast	0.57	0.29	0.72	0.60	0.45	0.42	0.30	0.29	0.67	0.11
km to Lakes or Streams	0.10	0.53	0.43	0.16	0.16	0.39	0.20	0.58	0.35	0.08
km to Major Road	0.38	0.32	0.33	0.35	0.28	0.34	0.20	0.52	0.53	0.26
km to Any Road	0.55	0.15	0.23	0.28	0.41	0.23	0.17	0.58	0.36	0.28
Annual Precipitation	0.46	0.29	0.46	0.30	0.48	0.47	0.10	0.74	0.62	0.20
Breeding Season Temp Range	0.32	0.23	0.65	0.36	0.19	0.45	0.06	0.68	0.58	0.29
Breeding Season Mean Temp	0.65	0.33	0.81	0.71	0.41	0.59	0.38	0.60	0.47	0.25
Canopy Cover	0.47	0.20	0.12	0.35	0.40	0.37	0.26	0.55	0.39	0.13
Tree Height	0.46	0.20	0.04	0.32	0.37	0.36	0.13	0.56	0.21	0.14
FPAR	0.55	0.47	0.14	0.45	0.51	0.23	0.31	0.67	0.38	0.31
Slope	0.41	0.38	0.13	0.36	0.30	0.09	0.09	0.41	0.22	0.14
Elevation	0.69	0.30	0.82	0.76	0.37	0.53	0.32	0.76	0.66	0.34
Heat Load Index	0.09	0.26	0.25	0.10	0.08	0.23	0.05	0.38	0.47	0.21

Pearson correlation coefficient for continuous environmental variables with bird sites at the three scales of the study (Klamath Ecoregion, vegetation formation, and park unit). See S2 Table for more information on environmental variables.

**Table 4:** Correlation of Bird Communities with Climate, Geography, and Vegetation.

	Biota/Environmental Matching			
	Spearman's ρ	No. of Variables	p Value	Variables maximizing correlation
Klamath Ecoregion	0.399	6	<0.001	Breeding Season Mean Temp, Breeding Season Temp Range, Canopy Cover, Elevation, FPAR, km to Any Road
Conifer	0.349	4	<0.001	Breeding Season Mean Temp, Breeding Season Temp Range, Elevation, FPAR
Mixed Conifer/Hardwood	0.338	4	<0.001	Annual Precipitation, Breeding Season Mean Temp, Breeding Season Temp Range, FPAR
Shrubland	0.399	5	<0.001	Annual Precipitation, Breeding Season Mean Temp, Canopy Cover, FPAR, km to Coast
Agriculture	0.645	1	<0.001	Elevation
Crater Lake National Park	0.247	6	0.011	Annual Precipitation, Breeding Season Mean Temp, Elevation, FPAR, km to Any Stream, km to Major Roads,
Lassen Volcanic National Park	0.224	6	0.013	Breeding Season Temp Range, Elevation, FPAR, km to Lake or Stream, km to Major Roads, Slope
Lava Beds National Monument	0.466	5	<0.001	Annual Precipitation, Breeding Season Mean Temp, Canopy Cover, km to Any Stream, km to Lake or Stream
Oregon Caves National Monument				
Redwood National and State Parks	0.472	6	0.008	Annual Precipitation, Canopy Cover, Elevation, km to Coast, km to Major Roads, Slope
Whiskeytown National Recreation Area	0.438	6	<0.001	Annual Precipitation, Breeding Season Mean Temp, Breeding Season Temp Range, Canopy Cover, Elevation, km to Any Stream

Subsets of environmental variables maximizing the correlation between bird sites and environmental variables (BIOENV). See methods for additional explanation and S2 Table for more information on environmental variables.

### Vegetation Formation

We associated each bird group with one of four broad vegetation formations in the Klamath Ecoregion: agriculture, conifer forest, mixed conifer/hardwood forest, and shrubland (S3 Table). Of the eight categorical variables included in analyses, the two broadest vegetation variables were most influential—environmental site potential and existing vegetation formation groups (Table 2). Bird groups in all vegetation formations were correlated with one or more continuous variables (Table 3).

**Fig 2:**
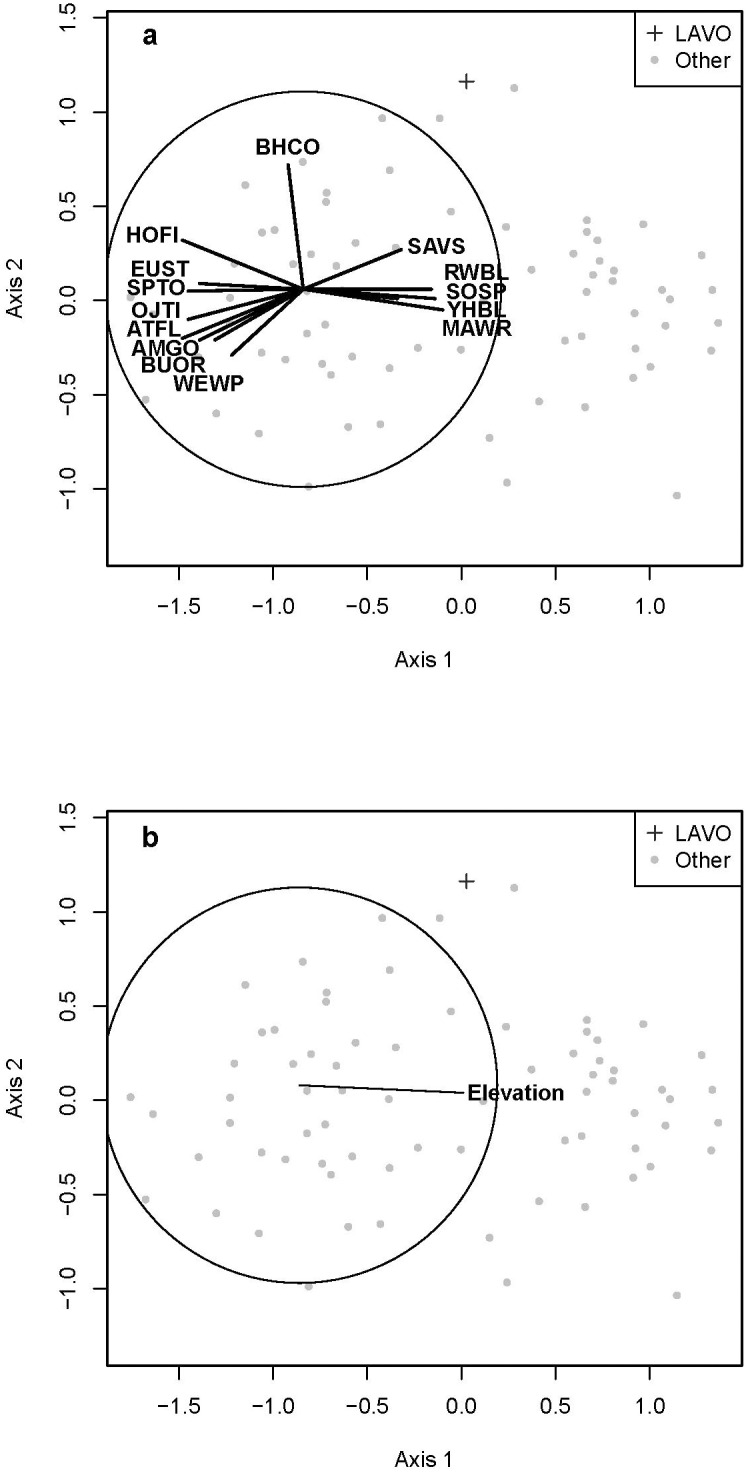
Bird Communities and Environmental Variables in Agriculture Habitat. Relationship of individual bird sites (displayed by park unit) identified as agriculture habitat (see text) using Non-metric Multidimensional Scaling. Biplots are direction and relative strength of Pearson correlation coefficients (the circle indicates a correlation of 1) between bird sites and (A) underlying bird species with a rho of > 0.5 and (B) environmental variables that maximized the overall correlation through BIOENV (Table 4). See S1 Table for bird codes and S2 Table for definitions and abbreviations of environmental variables.

The agriculture formation was the least sampled across the region; it included 80 sites (4%) and three bird groups. Of the eight categorical variables included in analyses, agriculture bird groups were significantly correlated with most, excluding existing vegetation formation broad classification, succession type, and disturbance category (Table 2). Only a single continuous environmental geography variable, elevation, was correlated to the species space gradient (Table 4), although weaker correlations with km to coast, breeding season temperature range, and mean temperature warrant noting (Table 3). Fourteen bird species drive the species space gradient associated with elevation (Fig 2a and 2b).

**Fig 3:**
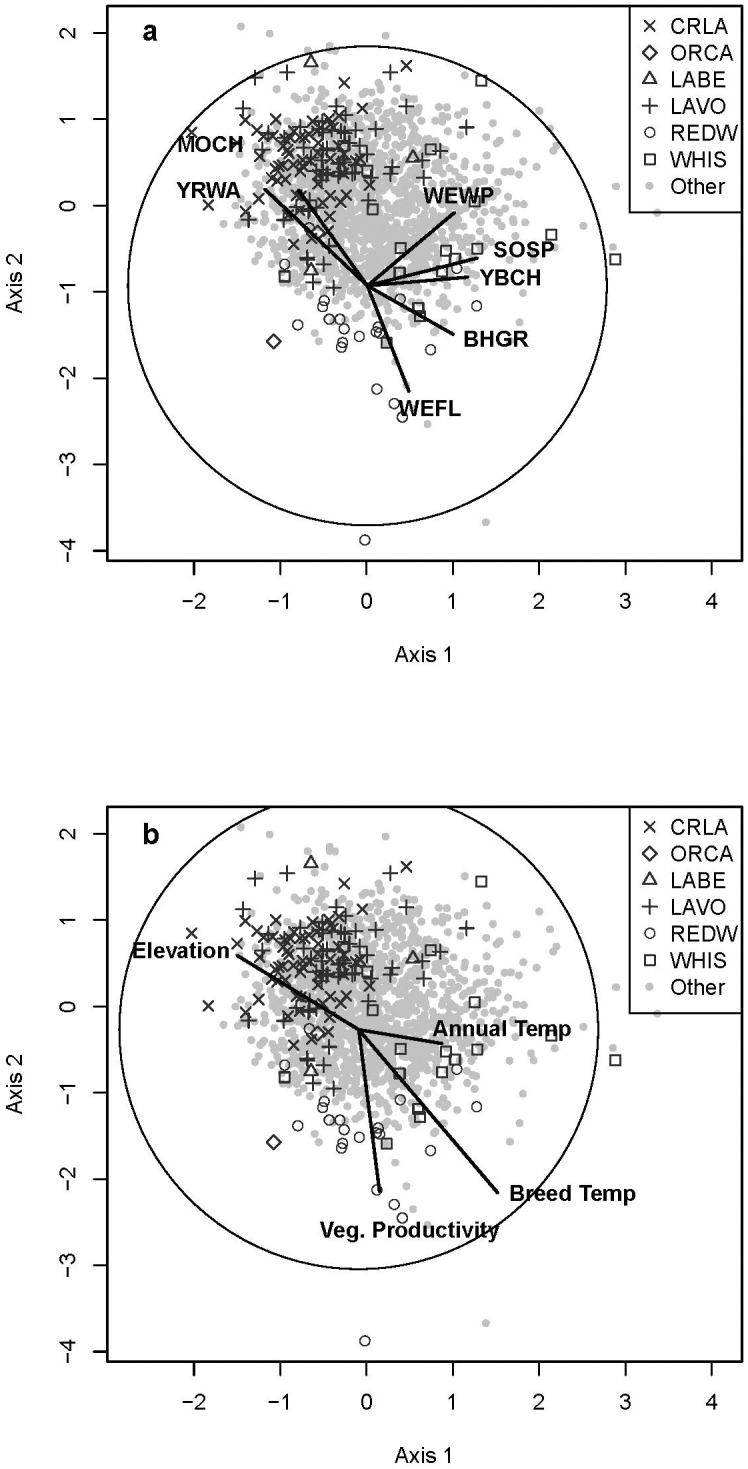
Bird Communities and Environmental Variables in Conifer Habitat. Relationship of individual bird sites (displayed by park unit) identified as conifer habitat (see text) using Non-metric Multidimensional Scaling. Biplots are direction and relative strength of Pearson correlation coefficients (the circle indicates a correlation of 1) between bird sites and (A) underlying bird species with a rho of > 0.5 and (B) environmental variables that maximized the overall correlation through BIOENV (Table 4). See S1 Table for bird codes and S2 Table for definitions and abbreviations of environmental variables.

The majority of study sites, 1411 of 1990 (90%), were located in the conifer forest formation. Fourteen of 29 bird groups were attributed to conifer forest. The two groups in conifer forest with the largest number of species, 76 and 77, included the greatest number of sites, 372 and 577 respectively, in combination making up 48% of our study sites (Table 1, S3 Table). Of the eight categorical variables, conifer forest bird groups were significantly correlated with all except disturbance category (Table 2). Four continuous environmental variables were best correlated with bird groups in conifer forest, two climate variables, one geography variable, and one vegetation variable (Tables 3 and 4). Seven bird species were the dominant drivers of two species space gradients associated with four environmental variables (Fig 3a and 3b).

**Fig 4:**
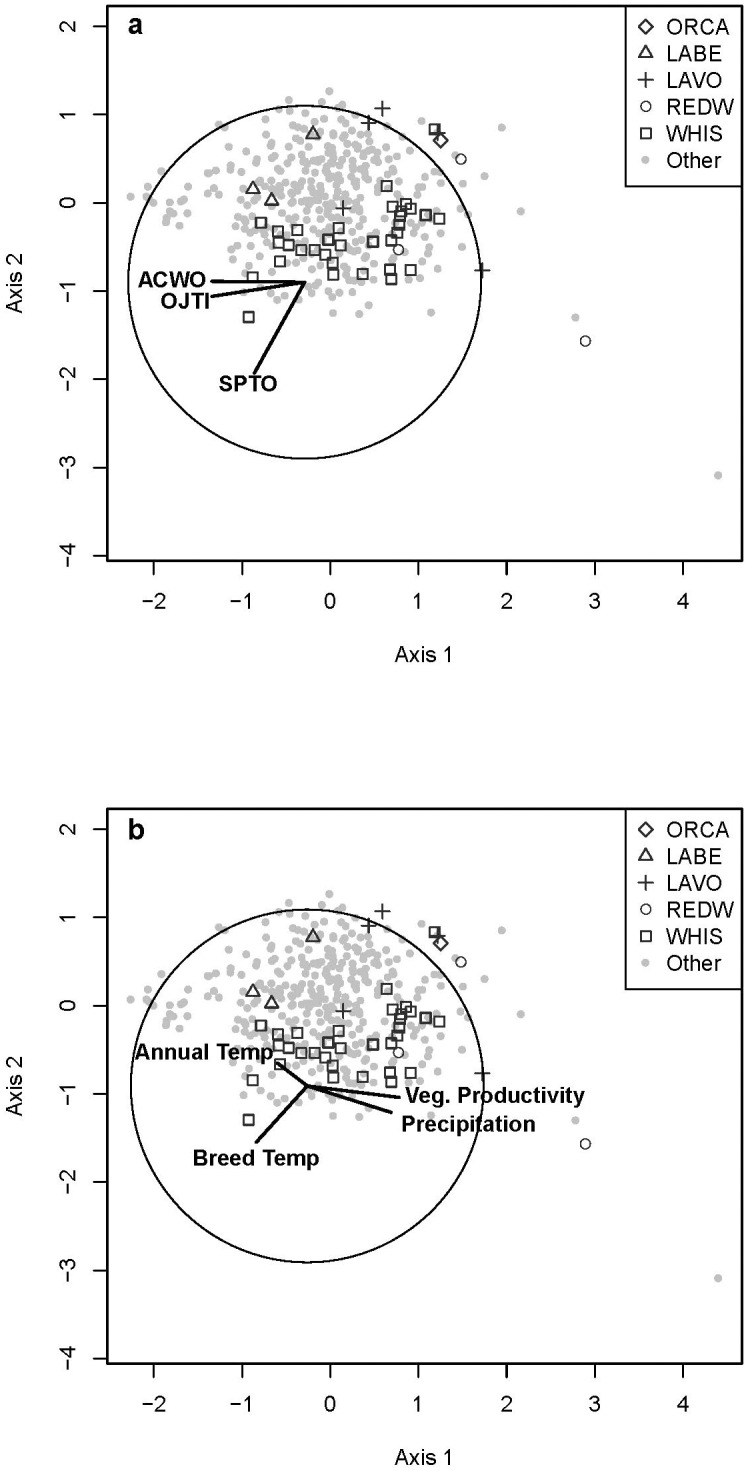
Bird Communities and Environmental Variables in Mixed Conifer-Hardwood Habitat. Relationship of individual bird sites (displayed by park unit) identified as mixed conifer-hardwood habitat (see text) using Non-metric Multidimensional Scaling. Biplots are direction and relative strength of Pearson correlation coefficients (the circle indicates a correlation of 1) between bird sites and (A) underlying bird species with a rho of > 0.5 and (B) environmental variables that maximized the overall correlation through BIOENV (Table 4). See S1 Table for bird codes and S2 Table for definitions and abbreviations of environmental variables.

The mixed conifer-hardwood formation made up the second greatest number of sites, 385 (19%), and included eight bird groups (Table 1, S3 Table). Of the eight categorical variables included in analyses, mixed conifer-hardwood forest bird groups were significantly correlated with most, excluding existing vegetation formation broad classification, aspect, and disturbance category (Table 2). Four continuous environmental variables were best correlated with bird groups in mixed conifer-hardwood forest, three climate variables, and one vegetation variable (Tables 3 and 4). Three bird species were the dominant drivers of two species space gradients associated with four environmental variables (Fig 4a and 4b).

**Fig 5:**
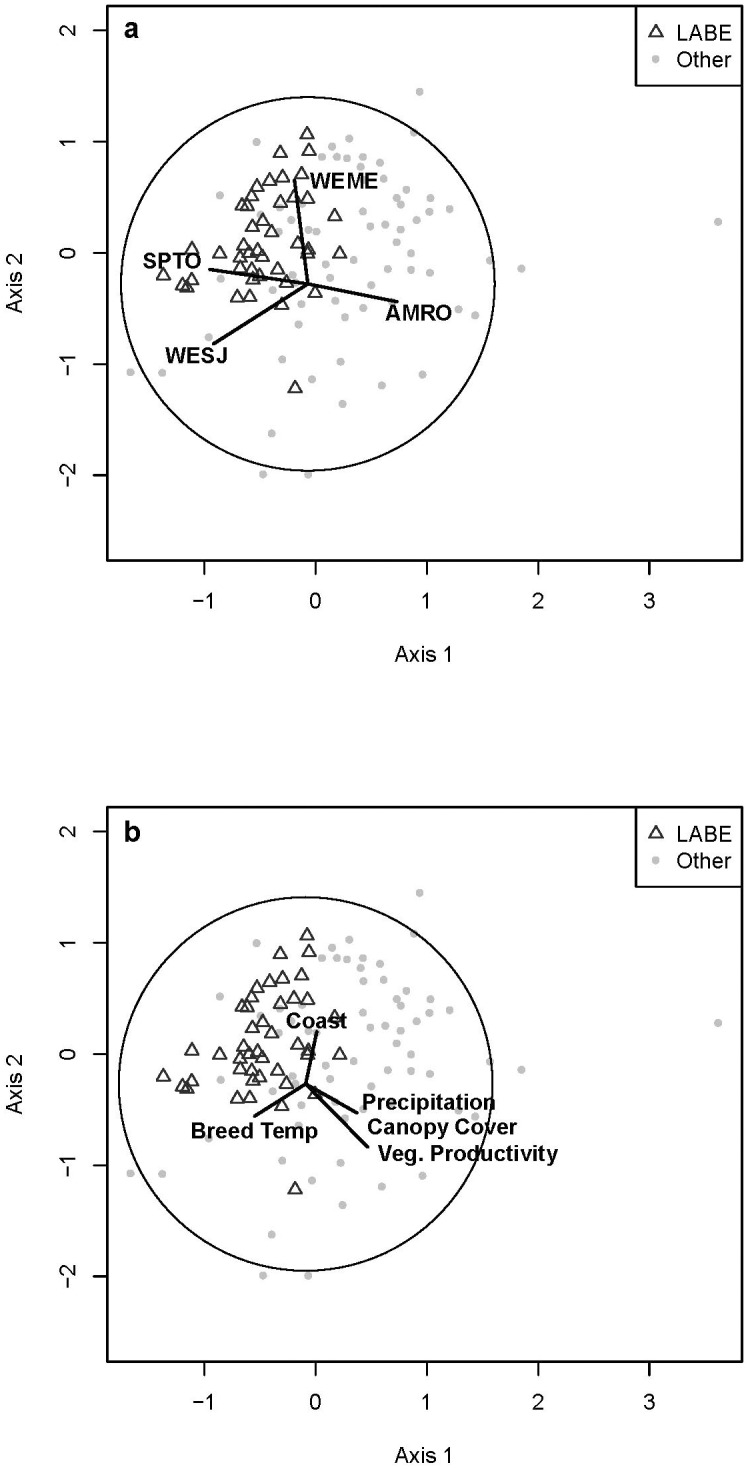
Bird Communities and Environmental Variables in Shrubland Habitat. Relationship of individual bird sites (displayed by park unit) identified as shrubland habitat (see text) using Non-metric Multidimensional Scaling. Biplots are direction and relative strength of Pearson correlation coefficients (the circle indicates a correlation of 1) between bird sites and (A) underlying bird species with a rho of > 0.5 and (B) environmental variables that maximized the overall correlation through BIOENV (Table 4). See S1 Table for bird codes and S2 Table for definitions and abbreviations of environmental variables.

The shrubland formation included 114 sites and four bird groups (S3 Table). Of the eight categorical variables, shrubland bird groups were significantly correlated with all except the disturbance category (Table 2). Five continuous environmental variables were best correlated with bird groups in shrubland, two climate variables, one geography variable, and two vegetation variables (Tables 3 and 4). Four bird species were the dominant drivers of two species space gradients associated with five environmental variables (Fig 5a and 5b).

### National Park Service Unit

Five of the six National Park Service units had large enough sample sizes to support meaningful analyses of statistically different bird groups. Oregon Caves NM did not have enough sites (only 3) to examine correlations with environmental variables, so for this park unit only bird groups were described. For the five park units included in analyses the two broadest vegetation variables were most influential, environmental site potential and existing vegetation formation groups of the eight categorical variables (Table 2). All five park units were correlated with continuous environmental variables (Tables 3 and 4). We provide park-specific findings below.

Crater Lake NP included six bird groups, two in conifer forest and four in mixed conifer/hardwood forest (S3 Table; Figs 3a and 4b). By far the greatest number of sites (56) was in the most common conifer forest bird group (group m in Table 1). Bird groups at Crater Lake NP were significantly correlated with environmental site potential, existing vegetation formation, and existing vegetation cover (Table 2). Six continuous environmental variables were best correlated; the strongest correlation was with breeding season mean temperature, although the relationship was only moderately strong (rho = 0.38) (Tables 3 and 4).

At Lassen Volcanic NP, like Crater Lake NP, the majority of sites were conifer forest (S3 Table), with the greatest number of points contributing to the most common bird group (group m in Table 1; Fig 3a and 3b). Nine additional conifer forest bird groups were present, along with 3 mixed-conifer/hardwood and one agricultural bird group. Bird groups at Lassen Volcanic NP were significantly correlated with environmental site potential, existing vegetation formation, and aspect (Table 2). Six continuous environmental variables were best correlated with bird communities, with the strongest correlation with km to any stream, although the relationship was not very strong (rho = 0.37) (Tables 3 and 4).

At Lava Beds NM, 88% of sites were shrubland (S3 Table), with 41 sites in a single bird group (group ag in Table 1; Fig 5a and 5b). There were also three conifer forest (Fig 3a and 3b) and two mixed conifer/hardwood bird groups (S3 Table; Fig 4a and 4b). Bird groups at Lava Beds NM were significantly correlated with environmental site potential, existing vegetation formation groups, existing vegetation formation broad classification, existing vegetation formation class, and disturbance (Table 2). Five continuous environmental variables were best correlated with bird groups at Lava Beds NM, with the strongest correlation to elevation (Tables 3 and 4).

There were only three sites in Oregon Caves NM and each was a distinct bird group; two were conifer forest (Fig 3a and 3b) and one was mixed conifer/hardwood forest (S3 Table, Fig 4a and 4b). As was noted above, because of its small size, this park unit did not have enough sites to analyze independently with environmental variables.

Redwood NSP had five bird groups, with the greatest number of sites (21) in one of the three conifer forest bird groups (group ac in Table 1; Fig 3a and 3b), and another two groups in mixed conifer/hardwood forest (S3 Table; Fig 4a and 4b). Bird groups at Redwood NSP were significantly correlated with environmental site potential category, existing vegetation formation groups, existing vegetation formation class, succession, aspect, and disturbance (Table 2). Six continuous environmental variables were best correlated with bird groups in this park unit, with the strongest correlations with km to coast and elevation (Tables 3 and 4).

Whiskeytown NRA included twelve bird groups: seven conifer forest (Fig 3a and 3b) and five mixed-conifer hardwood (S3 Table; Fig 4a and 4b). Bird groups at Whiskeytown NRA were significantly correlated with environmental site potential, existing vegetation formation groups, and disturbance (Table 2). The majority of sites contributed to two mixed-conifer hardwood bird groups (groups p and z in Table 1). Six continuous environmental variables were best correlated with bird groups at this park unit, with the strongest correlations being breeding season mean temperature and elevation (Tables 3 and 4).

## Discussion

We examined avian community ecology in the Klamath Ecoregion and determined that individual bird species co-exist spatially to form 29 bird groups, i.e. communities. Because patterns and processes of bird communities are scale dependent, we identified whether climate, geography, and vegetation variables were correlated to communities at three spatial scales: Klamath Ecoregion, vegetation formation, and National Park Service unit. Both continuous and categorical environmental variables were correlated with bird communities at all three spatial scales, suggesting that climate, geography, and vegetation interactively govern bird community distributions across the Klamath Ecoregion.

Two climate variables, breeding season mean temperature and temperature range, were correlated at all scales, but only with select vegetation formations and park units. Similarly, elevation, a geography variable, was correlated with all scales, but again only with select vegetation formations and park units. Although elevation was important at all scales, it is likely influencing birds differently based on the elevation range extent and extremes of a given scale. At the scale of the Klamath Ecoregion, elevation ranges from sea level to ~2000 meters; we would expect clearly differentiated bird communities across such an extreme biophysical gradient. In the north Cascades, Siegel et al. [51] found the narrowest elevation ranges for a small subset of species that were restricted to low or high elevation. We found elevation was not important in vegetation formations that tend to occur in a more narrow elevation band (i.e., mixed conifer/hardwood and shrubland).

Other geography variables differed by scale; aspect was important to three of four vegetation formations and two park units, and a greater number of geography variables were important at the park unit scale (e.g., km to any stream, km to lake or stream, slope). Two categorical vegetation variables, environmental site potential and existing vegetation formation groups were important at all spatial scales, vegetation formations, and park units. Canopy cover or vegetative productivity was also important at all scales, all vegetation formations with the exception of agriculture, and all park units. Additional vegetation variables became important at the scale of vegetation formation (i.e., succession) and park unit (i.e., disturbance).

### Klamath Ecoregion

As expected, we found bird community patterns with climate zones, geographical barriers, and vegetation formation within the Klamath Ecoregion [2,52]. Because of the complex geology of this region, it is likely that physical and biological controls interact, either continuously or abruptly, to create the spatial pattern of species composition and distribution. For birds, much of the landscape is effectively permeable and thus the mechanisms controlling geographic distributions appear to be individualistic and largely intangible (sensu [53]). Other studies have shown range boundaries to be correlated with temperature and other climate variables, but it is debatable whether such boundaries are direct climate limitations or differences associated with habitat, food resources, the presence of competitors, or some combination of all three. In a study of species distributions in the Eastern Andes Terborgh [54,55] found continuous elevation-driven, discrete (ecotonal), and competitive effects on biological distributions of individual species. In contrast, in a study of intermountain bird biogeography, Johnson [56] noted a rapid change in bird distributions east of the physiographic break of the Cascades-Sierra crest (at the east of the Klamath Region), yet a more gradual change across southern Nevada. He considered both to be climatically-driven gradients that were largely independent of competition [56]. We found that bird communities were organized along two gradients, one with elevation and breeding season mean temperature, explained largely by the heterogeneity of this region. A second gradient was correlated with canopy cover, vegetative productivity, and km to any road, suggesting that dense forest structure may be an important factor.

### Vegetation Formation

Elevation was the only variable correlated with agricultural bird communities, while vegetative productivity, canopy cover, and either breeding season mean temperature or temperature range were important for all other vegetation formations. The agriculture formation contained three bird communities. The community with the greatest number of sites (group ad) included five riparian and wetland species (Red-winged Blackbird, Song Sparrow, Yellow-headed Blackbird, Savanna Sparrow, Marsh Wren) and had high average similarity (46%) [57]. The next most common group (group v) had the greatest number of defining species of any community and the greatest average species per site (9.5). The species in that group were diverse, including those associated with riparian (Bullock’s Oriole, Yellow-breasted Chat), oak woodland (Oak or Juniper Titmouse, Downy Woodpecker, White-breasted Nuthatch), open shrubland (Spotted Towhee, Western Wood-pewee), and development or fragmentation (House Finch, Brown-headed Cowbird) [57]. House Finch and Brown-headed Cowbird also contributed to the third agriculture community (group t) along with two generalists (American Robin, House Wren) [57]. The bird communities suggest that the agricultural formation likely includes marsh habitat as well as farmland, and also that presence of individual bird species is likely influenced by adjacent edge habitats.

Within conifer sites, which were both the most abundant formation type, and characterized by the largest number of associated bird communities (14), habitat gradients were associated with elevation and breeding season mean temperature. The primary gradient for conifer formation was driven by bird species associated with elevation (e.g. Mountain Chickadee in groups k, l, m, and n) and breeding season mean temperature (e.g. Spotted Towhee in groups q, r, x, z and y). Among those communities there were distinct differences in bird species that can be attributed to forest conditions and habitat characteristics. The most common community (group m) included species that are indicative of conifer forest with mature overstory trees (e.g. Red-breasted Nuthatch, Hermit Warbler), but is missing species that require subcanopy or shrubs (e.g. Black-headed Grosbeak) [57]. In contrast, the second most common community (group r) includes species that use both mature overstory (e.g. Red-breasted Nuthatch, Hermit Warbler), as well as those that prefer subcanopy (e.g. Western Tanager, Cassin’s Vireo), and shrub components (e.g. Black-headed Grosbeak, Nashville Warbler, MacGillivray’s Warbler) [57]. Two bird communities include species associated with older trees and mature forest structure (i.e. large snags and downed wood) (e.g. Pacific Wren, Brown Creeper, Swainson’s Thrush); in combination they made up 4% of our study sites. The third most common conifer community (group y) includes species that use riparian deciduous subcanopy and shrubs (e.g. Song Sparrow, Bullock’s Oriole, Warbling Vireo) [57]. One of the communities (group j) with the fewest sites had five defining species, two of those riparian associates (Song Sparrow, MacGillivray’s Warbler) with a high average similarity (44%) [57]. These communities correlate with the primary conifer forest gradient and were positively correlated with breeding season mean temperature and breeding season temperature range, likely because areas of low elevation tend to include valley bottoms with riparian habitat and a warmer climate. Three conifer communities (groups h, q, x) were strongly driven by shrub associated species that prefer an open overstory (e.g. Olive-sided Flycatcher, Fox Sparrow, Western Wood-pewee, Lazuli Bunting) [57].

Bird communities in mixed conifer/hardwood forest fell along a gradient of vegetative productivity, annual precipitation, and breeding season temperature range. The species that contributed to those communities represent diverse components of mixed-conifer habitats ranging from conifer stands with a deciduous subcanopy in wetter, more maritime climates to pure oak woodlands in drier, more variable interior climates. The two communities that included the greatest number of sites (groups s and z) included species indicative of a deciduous subcanopy (e.g. Western Tanager, Western Wood-pewee) as well as a shrub component (Black-headed Grosbeak, Spotted Towhee) [57]. One of these groups included Black-throated Gray Warbler, a strong indicator of a robust deciduous subcanopy, often black oak (*Quercus kelloggii*) [57]. A high percentage of sites at Whiskeytown NRA were in mixed conifer/hardwood forest (groups p, r, and z) and they fell along the vegetative productivity and breeding season temperature gradient, also positively related to breeding season mean temperature. Areas where conifer and hardwood zones meet and overlap likely reflect a community that includes species unique to each. In a study in Spain, no species used only transitional oak-pine forest zones, rather these areas were inhabited by both oak and pine associated species, resulting in a slightly higher richness than either oak or pine forest alone [58]. Birds highly associated with oak woodland habitats (e.g. Oak or Juniper Titmouse, Acorn Woodpecker, White-breasted Nuthatch) were important in only a single group (group w) and had a relatively high average similarity (41%) [57]. This bird community was important in defining the gradient in mixed conifer/hardwood forest, with Acorn Woodpecker and Oak or Juniper Titmouse correlated with the ordination axes (Fig 4a). This highlights the importance of this unique, limited, and patchy vegetation formation. This is a vegetation formation that is projected to benefit from climate change in the Klamath Ecoregion [59].

The four communities in shrubland were spread along an environmental gradient associated with canopy cover and vegetative productivity, likely explained by a transition from shrubland to grassland. This is consistent with findings from Knick et al. [28], where a small set of habitat features were the primary drivers of shrub-steppe bird communities, interacting to a lesser extent with topography and geographic location. The greatest number of sites (group ag) was comprised of shrub (i.e. Spotted Towhee, Bewick’s Wren) and rock (i.e. Rock Wren) associated species. The second most common (group ah) was notable for its grassland associated species (e.g. Horned Lark, Vesper Sparrow, Savanna Sparrow) [57]. Grassland species (i.e. Western Meadowlark) at the low end of the canopy cover and vegetative productivity ordination gradient are among our most at risk species [57].

### National Park Service Units

At the scale of National Park Service units, we found correlations with a number of vegetation variables as expected [2]. However, we also found correlations with climate and geography, which we had not anticipated. Many of the climate and geography variables that were correlated at broader scales, such as breeding season mean temperature, annual precipitation, and elevation, were correlated with most of the park units, suggesting a range of conditions that is quite diverse even at this smaller scale. This is likely a reflection of the relatively high diversity of this region overall [19,22]. There were some correlations that were unique to the park unit scale; km to any stream or km to lake or stream were important for Crater Lake NP, Lassen Volcanic NP, Lava Beds NM, and Whiskeytown NRA. These geography variables were not important at the scale of the Klamath Ecoregion or for any vegetation formation. Disturbance was related to bird communities at Lava Beds NM and Whiskeytown NRA, but was not recognized in broader scale analyses.

There were no bird communities unique to the National Park Service units; all communities present in the park units occurred elsewhere in the Klamath Ecoregion. In contrast, seven communities did not occur in any park units, and six of those were relatively uncommon (i.e. comprised of less than 2% of sampling points). The oak woodland associated community (group w), which comprised 2.1% of total sites, was not present in the park unit sites suggesting this habitat type and associated bird community is under represented on National Park Service lands within the Klamath Ecoregion. Oak woodland occurs at Whiskeytown NRA and Redwood NSP, but in lesser proportions than in the larger region.

The community that is most heavily represented in the park units (group p) is a mixed conifer/hardwood community. Whiskeytown NRA included 37% of sites in this community. Whiskeytown NRA had 15 additional sites contributing to the similar, but shrubbier, community (group z). Both included Black-throated Gray Warbler, a strong indicator of deciduous subcanopy (often oak), and the latter including a number of shrub associated species as well. Lava Beds NM showed equal importance for shrubland communities, containing 61% of sites in the shrub and rock associated community and 12% of sites in the grassland community.

Sixteen percent of sites contributing to the most common community (group m) in conifer forest were located in park units, primarily at Crater Lake NP and Lassen Volcanic NP, with 56 and 38 sites respectively, but also present at Oregon Caves NM, Redwood NSP, and Whiskeytown NRA. In contrast, park units made up only 3% of the second most common community, also conifer, with a subcanopy and understory component suggesting a mid-elevation forest type that is not well represented in the park units, further supported by the clustering of Crater Lake NP and Lassen Volcanic NM with high elevation in the conifer formation. In contrast, a relatively uncommon conifer community which includes species associated with mature conifer forest in combination with a riparian component was found in three park units, making up 4 of the 11 sites in the Klamath Ecoregion. This indicates a strong representation of riparian environments in mature forest at Lassen Volcanic NP, Redwood NSP, and Whiskeytown NRA, an important contribution to this imperiled habitat type that is of particular significance to birds [60,61]. The community that contains species associated with mature forest structure is well represented in Redwood NSP, where 34% of sites were located, and was also present at Oregon Caves NM and Lassen Volcanic NP.

### Conclusion

We found 29 distinct bird communities in the Klamath Ecoregion, defined as individual suites of species co-occurring on the landscape at a given time [2,3]. Further study that explores functional differences among these communities is of interest and would have conservation implications beyond this work. In this study, we examined how patterns at three spatial scales (Klamath Ecoregion, vegetation formation, and National Park Service unit) correlated with gradients of climate, geography, and vegetation. Although we speculated that climate would be most important at the ecoregion scale, we found that two climate variables, breeding season mean temperature and temperature range, were correlated at all scales, suggesting that for some vegetation formations and park units there was sufficient variation in climate to be influential on bird communities. We expected vegetation variables to be most important at the park unit scale, but found broad metrics (environmental site potential and existing vegetation formation groups) meaningful across all scales; additional structural vegetation patterns were important at the scale of vegetation formation and park unit. The National Park Service units in the Klamath Ecoregion are inclusive of most bird communities with the exception of the oak woodland bird community; mature conifer forests are well represented, primarily associated with conifer canopy and lacking multi-layered structure. Overall, our results suggest that climate, geography, and vegetation interact across the Klamath Ecoregion, vegetation formations, and park units consistent with the high biodiversity of this region.

## Supporting Information

S1 Spreadsheet Bird presence/absence data. Presence/absence of songbird, woodpecker, and hummingbird species at 1,990 Klamath Ecoregion bird monitoring sites included in the final analyses. See S1 Table for common and scientific names.
(XLSX)

S2 Spreadsheet Environmental data. Environmental variables associated with 1,990 Klamath Ecoregion bird monitoring sites included in the final analyses. See S2 Table for data source, definitions, and scale.
(XLSX)

S1 Table List of Bird Species. List of bird species included in the study along with four letter codes, common names, and scientific names.
(DOCX)

S2 Table Environmental Variables. List of environmental variables included in the study along with the data source, definition, and scale of data.
(DOCX)

S3 Table Bird groups, vegetation formation, and park unit. Summary of statistically defined bird groups as determined with Similarity Profile (SimProf). “Group” is the arbitrary SimProf identifier, an asterisk indicates too few plots or species (<2) for inclusion in further analyses. Groups are classified into a single vegetation formation and the number of park unit sites that contribute to each group are included. See Table 1 and methods for more explanation.
(DOCX)
