# Supplementary material for: Bird Communities and Environmental Correlates in Southern Oregon and Northern California, USA
Source: PLoS One. 2016 Oct 12;11(10):e0163906. doi: 10.1371/journal.pone.0163906 (PMC5061419; doi:10.1371/journal.pone.0163906)
Supplement: S1 Table — List of bird species included in the study along with four letter codes, common names, and scientific names. (DOCX) [file pone.0163906.s003.docx]

**S1 Table. List of Bird Species.** List of bird species included in the study along with four letter codes, common and scientific names.

| Species Code | Common Name | Latin Name |
| --- | --- | --- |
| ACWO | Acorn Woodpecker | *Melanerpes formicivorus* |
| RBSA | Red-breasted Sapsucker | *Sphyrapicus ruber* |
| DOWO | Downy Woodpecker | *Picoides pubescens* |
| HAWO | Hairy Woodpecker | *Picoides villosus* |
| NOFL | Northern Flicker | *Colaptes auratus* |
| PIWO | Pileated Woodpecker | *Dryocopus pileatus* |
| OSFL | Olive-sided Flycatcher | *Contopus cooperi* |
| WEWP | Western Wood-Pewee | *Contopus sordidulus* |
| HAFL | Hammond's Flycatcher | *Empidonax hammondii* |
| GRFL | Gray Flycatcher | *Empidonax wrightii* |
| DUFL | Dusky Flycatcher | *Empidonax oberholseri* |
| WEFL | Pacific-slope or Cordilleran Flycatcher | *Empidonax difficilis or Empidonax occidentalis* |
| BLPH | Black Phoebe | *Sayornis nigricans* |
| ATFL | Ash-throated Flycatcher | *Myiarchus cinerascens* |
| WEKI | Western Kingbird | *Tyrannus verticalis* |
| CAVI | Cassin's Vireo | *Vireo cassinii* |
| HUVI | Hutton's Vireo | *Vireo huttoni* |
| WAVI | Warbling Vireo | *Vireo gilvus* |
| STJA | Steller's Jay | *Cyanocitta stelleri* |
| WESJ | Western Scrub-Jay | *Aphelocoma californica* |
| GRAJ | Gray Jay | *Perisoreus canadensis* |
| CLNU | Clark's Nutcracker | *Nucifraga columbiana* |
| BBMA | Black-billed Magpie | *Pica hudsonia* |
| AMCR | American Crow | *Corvus brachyrhynchos* |
| CORA | Common Raven | *Corvus corax* |
| HOLA | Horned Lark | *Eremophila alpestris* |
| VGSW | Violet-green Swallow | *Tachycineta thalassina* |
| NRWS | Northern Rough-winged Swallow | *Stelgidopteryx serripennis* |
| CLSW | Cliff Swallow | *Petrochelidon pyrrhonota* |
| BARS | Barn Swallow | *Hirundo rustica* |
| BCCH | Black-capped Chickadee | *Poecile atricapillus* |
| MOCH | Mountain Chickadee | *Poecile gambeli* |
| CBCH | Chestnut-backed Chickadee | *Poecile rufescens* |
| OJTI | Oak or Juniper Titmouse | *Baeolophus inornatus or Baeolophus ridgwayi* |
| BUSH | Bushtit | *Psaltriparus minimus* |
| RBNU | Red-breasted Nuthatch | *Sitta canadensis* |
| WBNU | White-breasted Nuthatch | *Sitta carolinensis* |
| BRCR | Brown Creeper | *Certhia americana* |
| ROWR | Rock Wren | *Salpinctes obsoletus* |
| CANW | Canyon Wren | *Catherpes mexicanus* |
| BEWR | Bewick's Wren | *Thryomanes bewickii* |
| HOWR | House Wren | *Troglodytes aedon* |
| PAWR | Pacific Wren | *Troglodytes pacificus* |
| MAWR | Marsh Wren | *Cistothorus palustris* |
| GCKI | Golden-crowned Kinglet | *Regulus satrapa* |
| RCKI | Ruby-crowned Kinglet | *Regulus calendula* |
| WEBL | Western Bluebird | *Sialia mexicana* |
| TOSO | Townsend's Solitaire | *Myadestes townsendi* |
| SWTH | Swainson's Thrush | *Catharus ustulatus* |
| HETH | Hermit Thrush | *Catharus guttatus* |
| AMRO | American Robin | *Turdus migratorius* |
| VATH | Varied Thrush | *Ixoreus naevius* |
| WREN | Wrentit | *Chamaea fasciata* |
| SATH | Sage Thrasher | *Oreoscoptes montanus* |
| CATH | California Thrasher | *Toxostoma redivivum* |
| EUST | European Starling | *Sturnus vulgaris* |
| OCWA | Orange-crowned Warbler | *Vermivora celata* |
| NAWA | Nashville Warbler | *Oreothlypis ruficapilla* |
| YEWA | Yellow Warbler | *Setophaga petechia* |
| YRWA | Yellow-rumped Warbler | *Dendroica coronata* |
| BTYW | Black-throated Gray Warbler | *Dendroica nigrescens* |
| HEWA | Hermit Warbler | *Dendroica occidentalis* |
| MGWA | MacGillivray's Warbler | *Oporornis tolmiei* |
| COYE | Common Yellowthroat | *Geothlypis trichas* |
| WIWA | Wilson's Warbler | *Wilsonia pusilla* |
| YBCH | Yellow-breasted Chat | *Icteria virens* |
| GTTO | Green-tailed Towhee | *Pipilo chlorurus* |
| SPTO | Spotted Towhee | *Pipilo maculatus* |
| CALT | California Towhee | *Pipilo crissalis* |
| CHSP | Chipping Sparrow | *Spizella passerina* |
| BRSP | Brewer's Sparrow | *Spizella breweri* |
| VESP | Vesper Sparrow | *Pooecetes gramineus* |
| LASP | Lark Sparrow | *Chondestes grammacus* |
| SAVS | Savannah Sparrow | *Passerculus sandwichensis* |
| FOSP | Fox Sparrow | *Passerella iliaca* |
| SOSP | Song Sparrow | *Melospiza melodia* |
| WCSP | White-crowned Sparrow | *Zonotrichia leucophrys* |
| GCSP | Golden-crowned Sparrow | *Zonotrichia atricapilla* |
| DEJU | Dark-eyed Junco | *Junco hyemalis* |
| BHGR | Black-headed Grosbeak | *Pheucticus melanocephalus* |
| LAZB | Lazuli Bunting | *Passerina amoena* |
| RWBL | Red-winged Blackbird | *Agelaius phoeniceus* |
| WEME | Western Meadowlark | *Sturnella neglecta* |
| YHBL | Yellow-headed Blackbird | *Xanthocephalus* |
| BRBL | Brewer's Blackbird | *Euphagus cyanocephalus* |
| BHCO | Brown-headed Cowbird | *Molothrus ater* |
| BUOR | Bullock's Oriole | *Icterus bullockii* |
| RECR | Red Crossbill | *Loxia curvirostra* |
| PISI | Pine Siskin | *Spinus pinus* |
| LEGO | Lesser Goldfinch | *Spinus psaltria* |
| AMGO | American Goldfinch | *Spinus tristis* |
| HOSP | House Sparrow | *Passer domesticus* |
| WETA | Western Tanager | *Piranga ludoviciana* |
| PUFI | Purple Finch | *Carpodacus purpureus* |
| CAFI | Cassin's Finch | *Carpodacus cassinii* |
| HOFI | House Finch | *Carpodacus mexicanus* |
