# Supplementary material for: Bird Communities and Environmental Correlates in Southern Oregon and Northern California, USA
Source: PLoS One. 2016 Oct 12;11(10):e0163906. doi: 10.1371/journal.pone.0163906 (PMC5061419; doi:10.1371/journal.pone.0163906)
Supplement: S2 Table — List of environmental variables included in the study along with the data source, definition, and scale of data. (DOCX) [file pone.0163906.s004.docx]

**S2 Table. Environmental Variables.** List of environmental variables included in the study along with the data source and scale of data.

| Variable [Abbreviation] | Short Description | Source | Scale | Units |
| --- | --- | --- | --- | --- |
| Aspect | Compass bearing category (N, NW, etc.) | DEM | 30m | Categorical |
| Elevation | Height above sea level | DEM | 30m | m |
| Slope | Percent change of elevation over a specific area | DEM | 30m | degrees |
| Canopy Cover | Estimated Canopy Cover % | Landfire | 30m | % |
| Disturbance | Type of Disturbance | Landfire | 30m | Categorical |
| Environmental Site Potential | Represents vegetation that could be supported at a given site based on the biophysical environment | Landfire | 30m | Categorical |
| Existing Vegetation Cover | Vertically projected percent cover of live canopy | Landfire | 30m | Categorical |
| Existing Vegetation Type | Species composition currently present at a site | Landfire | 30m | Categorical |
| Existing Vegetation Type Broad Classification | Species composition currently present at a site, group physiognomy | Landfire | 30m | Categorical |
| Existing Vegetation Type Group Name | Aggregated existing vegetation types based on dominant species or groups of dominant species | Landfire | 30m | Categorical |
| Succession Class | Current vegetation conditions with respect to the vegetation species composition, cover, and height ranges of successional states that occur within each biophysical setting | Landfire | 30m | Categorical |
| Tree Height | The average height of the top of the vegetated canopy | Landfire | 30m | m |
| km to Any Road | Distance to any road, paved or dirt | NPScape | 1:24,000 | km |
| km to Major Road | Distance to roads with an ESRI feature class code of A20-A38, excluding ferry routes | NPScape | 1:24,000 | km |
| km to Coast [Coast] | Distance to the Pacific Ocean coastline | NPScape | 1:24,000 | km |
| Annual Precipitation [Precipitation] | 30 year annual normal precipitation | PRISM | 800m | mm |
| Breeding Season Avg Temp (4 to 7) [Breed Temp] | 30 year normal mean temperature for the months of April - July | PRISM | 800m | °C |
| Temp Range (4 to 7) [Annual Temp] | Average difference between the max and min 30 year normal temperature during the months of April-July | PRISM | 800m | °C |
| km to Any Stream | Distance to a perennial or intermittent stream | USGS NHD | 1:24,000 | km |
| km to Waterbodies | Distance to basic water bodies are included here such as lakes, ponds, and reservoirs. | USGS NHD | 1:24,000 | km |
| FPAR [Veg. Productivity] | Fraction of absorbed PAR | USGS-MODIS | 1km | unitless |
| Heat Load Index | An index of heat load taking into account aspect, elevation, and latitude | DEM/Landfire | 30m | unitless |
