# Supplementary material for: Bird Communities and Environmental Correlates in Southern Oregon and Northern California, USA
Source: PLoS One. 2016 Oct 12;11(10):e0163906. doi: 10.1371/journal.pone.0163906 (PMC5061419; doi:10.1371/journal.pone.0163906)
Supplement: S3 Table — Summary of statistically defined bird groups as determined with Similarity Profile (SimProf). “Group” is the arbitrary SimProf identifier, an asterisk indicates too few plots or species (<2) for inclusion in further analyses. Groups are classified into a single vegetation formation and the number of park unit sites that contribute to each group are included. See Table 1 and methods for more explanation. (DOCX) [file pone.0163906.s005.docx]

**S3 Table. Bird groups, vegetation formation, and park unit.** Summary of statistically defined bird groups as determined with Similarity Profile (SimProf). “Group” is the arbitrary SimProf identifier, an asterisk indicates too few plots or species (<2) for inclusion in further analyses. Groups are classified into a single vegetation formation and the number of park unit sites that contribute to each group are included. See Table 1 and methods for more explanation.

|  | Vegetation Formation | National Park Service Park Unit | | | | | |
| --- | --- | --- | --- | --- | --- | --- | --- |
| Group |  | CRLA | LABE | LAVO | ORCA | REDW | WHIS |
| a | * | * | * | * | * | * | * |
| b | * | * | * | * | * | * | * |
| c | * | * | * | * | * | * | * |
| d | Conifer | 3 | 0 | 2 | 0 | 0 | 0 |
| e | Mixed Conifer/Hardwood | 0 | 0 | 2 | 0 | 1 | 3 |
| f | Mixed Conifer/Hardwood | 0 | 0 | 0 | 1 | 0 | 0 |
| g | Conifer | 0 | 0 | 1 | 0 | 0 | 1 |
| h | Conifer | 0 | 0 | 2 | 0 | 0 | 1 |
| i | Conifer | 1 | 1 | 5 | 0 | 0 | 1 |
| j | Conifer | 0 | 0 | 0 | 0 | 0 | 0 |
| k | Conifer | 1 | 0 | 0 | 0 | 0 | 0 |
| l | Conifer | 2 | 0 | 6 | 0 | 0 | 0 |
| m | Conifer | 56 | 0 | 38 | 1 | 1 | 1 |
| n | Conifer | 1 | 1 | 3 | 0 | 0 | 0 |
| o | Mixed Conifer/Hardwood | 0 | 0 | 0 | 0 | 0 | 0 |
| p | Mixed Conifer/Hardwood | 0 | 0 | 0 | 0 | 0 | 13 |
| q | Conifer | 0 | 0 | 0 | 0 | 0 | 1 |
| r | Conifer | 0 | 1 | 1 | 0 | 2 | 9 |
| s | Mixed Conifer/Hardwood | 0 | 1 | 2 | 0 | 0 | 1 |
| t | Agriculture | 0 | 0 | 1 | 0 | 0 | 0 |
| u | Shrubland | 0 | 0 | 0 | 0 | 0 | 0 |
| v | Agriculture | 0 | 0 | 0 | 0 | 0 | 0 |
| w | Mixed Conifer/Hardwood | 0 | 0 | 0 | 0 | 0 | 0 |
| x | Conifer | 0 | 0 | 2 | 0 | 0 | 0 |
| y | Conifer | 0 | 0 | 0 | 0 | 0 | 4 |
| z | Mixed Conifer/Hardwood | 0 | 2 | 0 | 0 | 0 | 15 |
| aa |  | * | * | * | * | * | * |
| ab | Mixed Conifer/Hardwood | 0 | 0 | 1 | 0 | 2 | 1 |
| ac | Conifer | 0 | 0 | 2 | 1 | 21 | 0 |
| ad | Agriculture | 0 | 0 | 0 | 0 | 0 | 0 |
| ae | * | * | * | * | * | * | * |
| af | Shrubland | 0 | 0 | 0 | 0 | 0 | 0 |
| ag | Shrubland | 0 | 41 | 0 | 0 | 0 | 0 |
| ah | Shrubland | 0 | 4 | 0 | 0 | 0 | 0 |
